# Discovery of a novel long noncoding RNA overlapping the *LCK* gene that regulates prostate cancer cell growth

**DOI:** 10.1186/s12943-019-1039-6

**Published:** 2019-06-28

**Authors:** Huy Q. Ta, Hilary Whitworth, Yi Yin, Mark Conaway, Henry F. Frierson, Moray J. Campbell, Ganesh V. Raj, Daniel Gioeli

**Affiliations:** 1 Departments of Microbiology Immunology, and Cancer Biology, University of Virginia, Charlottesville, Virginia 22908 USA; 2 Cancer Center Member, University of Virginia, Charlottesville, Virginia USA; 3 Department of Pathology, University of Virginia Health System, Charlottesville, Virginia USA; 4 Department of Public Health Sciences, University of Virginia, Charlottesville, Virginia USA; 5 College of Pharmacy Pharmaceutics and Pharmaceutical Chemistry, The Ohio State University, Columbus, OH 43210 USA; 6 Department of Urology, UT Southwestern Medical Center, Dallas, TX 75390 USA

**Keywords:** HULLK, Long noncoding RNA, Prostate cancer, Androgen receptor, LCK

## Abstract

**Background:**

Virtually all patients with metastatic prostate cancer (PCa) will relapse and develop lethal castration-resistant prostate cancer (CRPC). Long noncoding RNAs (lncRNAs) are emerging as critical regulatory elements of many cellular biological processes, and may serve as therapeutic targets for combating PCa progression. Here, we have discovered in a high-throughput RNAi screen a novel lncRNA in PCa, and assessed the oncogenic effects of this lncRNA.

**Methods:**

Rapid amplification of cDNA ends and sequencing was utilized to identify a previously unannotated lncRNA lying within exon six and the 3’UTR of the *lymphocyte-specific protein tyrosine kinase* (*LCK)* gene. The levels of HULLK in the presence or absence of hormone and/or enzalutamide or coregulator inhibitors were measured by quantitative PCR (qPCR). The determination of HULLK transcription and localization were characterized by strand-specific qPCR and cellular fractionation followed by qPCR, respectively. The correlation between HULLK expression and prostate cancer Gleason score was analyzed by droplet digital PCR. CyQuant assays were conducted to evaluate the effects of knocking down HULLK with shRNAs or overexpressing HULLK on cell growth.

**Results:**

In this study, a previously unannotated lncRNA lying within exon six and 3’UTR of the *LCK* gene was dramatically upregulated by androgen in a dose-dependent manner, and the anti-androgen enzalutamide completely blocked this hormone-induced increase. Therefore, we labeled this lncRNA “HULLK” for Hormone-Upregulated lncRNA within *LCK*. Binding sites for two AR coregulators p300 and Brd4 reside near the HULLK transcriptional start site (TSS), and inhibitors of these coregulators downregulated HULLK. HULLK is transcribed from the sense strand of DNA, and predominantly localizes to the cytoplasm. HULLK transcripts are not only expressed in prostate cancer cell lines, but also prostate cancer patient tissue. Remarkably, there was a significant positive correlation between HULLK expression and high-grade PCa in multiple cohorts. shRNAs targeting HULLK significantly decreased PCa cell growth. Moreover, cells overexpressing HULLK were hypersensitive to androgen stimulation.

**Conclusions:**

HULLK is a novel lncRNA situated within the *LCK* gene that may serve as an oncogene in PCa. Our data enhances our understanding of lncRNA biology and may assist in the development of additional biomarkers or more effective therapeutic targets for advanced PCa.

**Electronic supplementary material:**

The online version of this article (10.1186/s12943-019-1039-6) contains supplementary material, which is available to authorized users.

## Background

Next-generation sequencing of the PCa transcriptome has uncovered that approximately a quarter of abundant transcripts are lncRNAs, suggesting that they may play a larger role in PCa than originally thought [1]. In fact, PCa-specific lncRNAs have been reported for every stage in the progression of the disease. Therefore, lncRNAs may serve as therapeutic targets for combating PCa progression.

In hormone-sensitive PCa, long noncoding RNA Activated by Transforming Growth Factor-Beta (lncRNA-ATB) upregulates the expression of epithelial-to-mesenchymal transition (EMT) factors, which is an important component that promotes CRPC [2]. Furthermore, lncRNA-ATB overexpression increases proliferation through the elevation in cyclin D1 and cyclin E. In addition to playing a role in EMT, Prostate Cancer Antigen 3 (PCA3) modulates the expression of several cancer-related genes (vascular endothelial growth factor A, receptor tyrosine-protein kinase, Bcl-2-associated death promoter, and telomerase reverse transcriptase) and androgen receptor (AR) cofactors [3]. PCA3 also regulates tumor suppressor prune homolog 2 (*PRUNE2*) expression by forming a PRUNE2/PCA3 double-stranded RNA complex which results in PRUNE2 translational repression [4]. The lncRNA Differentiation Antagonizing Non-Protein Coding RNA (DANCR) has been shown to counter the actions of the androgen-AR signaling axis [5], which drives epithelial cell terminal differentiation in the normal prostate [6] and inhibits invasion and metastasis in PCa [7]. Probably functioning as a scaffold, DANCR suppresses differentiation and promotes invasion and metastasis by negatively regulating tissue inhibitor of metalloproteinases 2/3 (TIMP 2/3) expression through enhancer of zeste homolog 2 recruitment [8]. The androgen-AR signaling pathway is also opposed by Prostate Cancer-Associated Transcript 29 (PCAT29), which inhibits proliferation and is repressed by the AR [9]. Thus, PCa progression may be driven by these lncRNAs at the hormone-sensitive stage of the disease.

During androgen deprivation therapy (ADT), there are two lncRNAs that have been reported to be overexpressed and promote progression to CRPC. Prostate Cancer Gene Expression Marker 1, elevated in response to ADT, translocates to the nucleus and binds U2 small nuclear RNA auxillary factor 2 and heterogeneous nuclear ribonucleoprotein A1. This results in the upregulation of AR splice variant 7 (AR-V7), and ultimately, induces proliferation [10]. C-terminal Binding Protein 1-Antisense (CTBP1-AS) inhibits CTBP1 expression by complexing with the transcriptional repressor Polypyrimidine Tract Binding Protein (PTB)-associating splicing factor (PSF) and recruiting histone decarboxylase (HDAC)-paired amphipathic helix protein Sin3a (Sin3A) complexes to the gene promoter [11]. Furthermore, CTBP1-AS promotes cell cycle progression and proliferation through the suppression of p53 and mothers against decapentaplegic homolog 3 expression. Thus, these two lncRNAs may drive CRPC.

While many lncRNAs have been implicated in PCa progression to CRPC, only a small subset has been experimentally validated. For example, the proliferative and invasive capacities of CRPC cells are enhanced as a result of the overexpression of HOX Transcript Antisense RNA (HOTAIR) [12]. While the AR usually decreases HOTAIR expression, HOTAIR increases AR activity by preventing ubiquitination and degradation of the AR through the inhibition of mouse double minute 2 homolog (MDM2). The estrogen receptor α (ERα)-regulated lncRNA Nuclear-Enriched Abundant Transcript 1 (NEAT1) not only controls the levels of specific PCa genes, but it also modulates the expression of the Transmembrane Protease, Serine 2 (TMPRSS2)-ERG fusion gene [13]. Moreover, ERG-positive CRPC frequently overexpresses PCAT5 [14]. Depletion of PCAT5 from PC3 cells diminishes proliferation and invasion and induces apoptosis. Similar to PCAT5, Second Chromosome Locus Associated with Prostate-1 (SChLAP1) also correlates with ERG-positive PCa [15]. SChLAP1, which is overexpressed in approximately 25% of all PCa, promotes invasion and migration by interacting with the SWItch/Sucrose Non-Fermentable-complex and blocking its gene expression regulatory function. Despite the growing list of annotated lncRNAs predicted by RNA sequencing, the number of lncRNAs that have been thoroughly defined and experimentally verified is small overall. It is presumable that thousands of lncRNAs remain to be detected since those arising from overlapping protein-coding loci still need to be analyzed [16].

Here, we have discovered that there is a novel lncRNA encoded within the *lymphocyte-specific protein tyrosine kinase* (*LCK)* gene locus in PCa cells. We illustrate that this lncRNA is regulated by the AR, as expression was increased in response to hormone and blocked in the presence of enzalutamide, as well as inhibitors of p300 and the bromodomain and extra-terminal (BET) family. As a result, we have labelled this lncRNA “HULLK” for Hormone Upregulated lncRNA within *LCK*. It is transcribed from the sense strand of DNA and localizes to the cytoplasm. HULLK transcripts are not only expressed in PCa cell lines, but also PCa patient tissue. Furthermore, we found that HULLK expression is significantly upregulated with increasing tumor grade. We demonstrated that HULLK knockdown with shRNAs significantly decrease cellular proliferation in the presence and absence of hormone. HULLK overexpression hypersensitizes PCa cells to androgen. Thus, our data indicates that HULLK is a lncRNA situated within the *LCK* gene that functions as a novel positive regulator of PCa cell growth.

## Methods

### Cell culture and reagents

LNCaP and C4–2 cells (a gift from Dr. L. W. K. Chung) were grown in DMEM:F12 (Invitrogen) with 5% Non-Heat-Inactivated serum (Gemini) and 1% Insulin-Transferrin-Selenium-Ethanolamine (ThermoFisher). CWR22Rv1 (Rv1), VCaP, PC3 (gifts from Drs. Steven Balk, Karen Knudsen, and Chung, respectively), WMPY-1 (ATCC), MCF7, BT549 and MDA-MB-231 (gifts from Dr. Amy Bouton) were grown in DMEM (Invitrogen) with 10% Heat-Inactivated serum. DU145 (a gift from Dr. Chung), PANC1 (a gift from Dr. J. Thomas Parsons), HeLa and Jurkat cells (gifts from Dr. Tim Bender) were grown in RPMI-1640 (Invitrogen) with 10% Heat-Inactivated serum. LHS cells (a gift from Dr. William Hahn) were grown in ProstaLife Epithelial Cell Medium (Lifeline). RWPE-1 (ATCC) cells were grown in Keratinocyte-Serum-Free Media (Invitrogen). For growth and RNA experiments, phenol-red free DMEM:F12 media with 5% Charcoal-Stripped Serum (CSS) (Gemini) was used.

Antibodies: ERK1/2 (137F5), Histone H3, LCK (73A5) (c-term), p53 (DO-7), Ran, α-Tubulin (Cell Signaling); LCK (n-term) (BD Biosciences); AR (in-house to first 21aa). Western blotting performed as previously described [17, 18].

HULLK primers were based on the human LCK sequence obtained from Genbank (BC013200.1) (Additional file 1: Figure S1). PCR was performed using iProof High-Fidelity DNA Polymerase (Bio-Rad) and LNCaP cDNA as the template. HULLK was ligated into the lentiviral expression vector PLX301 (a gift from David Root, Addgene plasmid #25895) using Gateway Cloning (ThermoFisher), transformed into DH5α competent bacteria (Life Technologies), and clones were sequenced for verification.

### CyQuant growth assays

Assay was performed as previously described [18]. Briefly, shRNA or pLKO control virus was added to 1 μg/ml fibronectin-coated 96well plates. Cells were plated in RPMI-1640 plus 5%CSS with vehicle, 0–0.05 nM R1881, and/or 10 μM Enzalutamide (Selleck Chemicals). Quantification was performed on Day 7 using a BioTek Synergy 2 plate reader.

### Rapid amplification of cDNA ends (RACE)

5′ and 3′ RACE was performed according to the manufacturer’s protocol (Invitrogen) using an anti-sense primer specific for LCK exon 11 (5′ RACE) and a sense primer for LCK exon 9 (3′ RACE). 5′/3′ RACE PCR products were ligated into the pGEM sequencing vector, and multiple clones were sequenced and aligned using the UCSC genome browser.

### RNA isolation, qPCR and ddPCR

RNA was collected using TRIzol and quantitated using a NanoDrop 2000 UV-Vis Spectrophotometer (ThermoFisher). cDNA was synthesized using Superscript IV VILO cDNA synthesis kit (Invitrogen). Quantitative real-time PCR (qPCR) was performed as previously described [17, 18]. Droplet digital PCR (ddPCR) was executed according to the manufacturer’s protocol (Bio-Rad).

### Patient samples

Formalin-fixed paraffin-embedded (FFPE) tissue was minced and deparaffinized prior to RNA isolation. Fresh-frozen tissue was homogenized with a mortar and pestle prior to RNA collection.

### TCGA prostate cancer cohort

The LCK exon-specific RSEM data for all samples in TCGA-PRAD cohort [19] was obtained from TSVdb [20] and the sum of RSEM values in exons 9–12 was compared to exons 1–3 to determine a 3′/5′ ratio and define HULLK expression as an increase in the 3′/5′ ratio. Clinical data for the TCGA-PRAD cohort was down loaded from cBioPortal and LCK 3′/5′ ratio was examined comparing normal to tumor and, in tumors only, low Gleason [scores 6 and 7(3 + 4)] to high Gleason score [scores 7(4 + 3), and 8–10].

## Results

### Discovery of a novel lncRNA in PCa cells

**Fig. 1:**
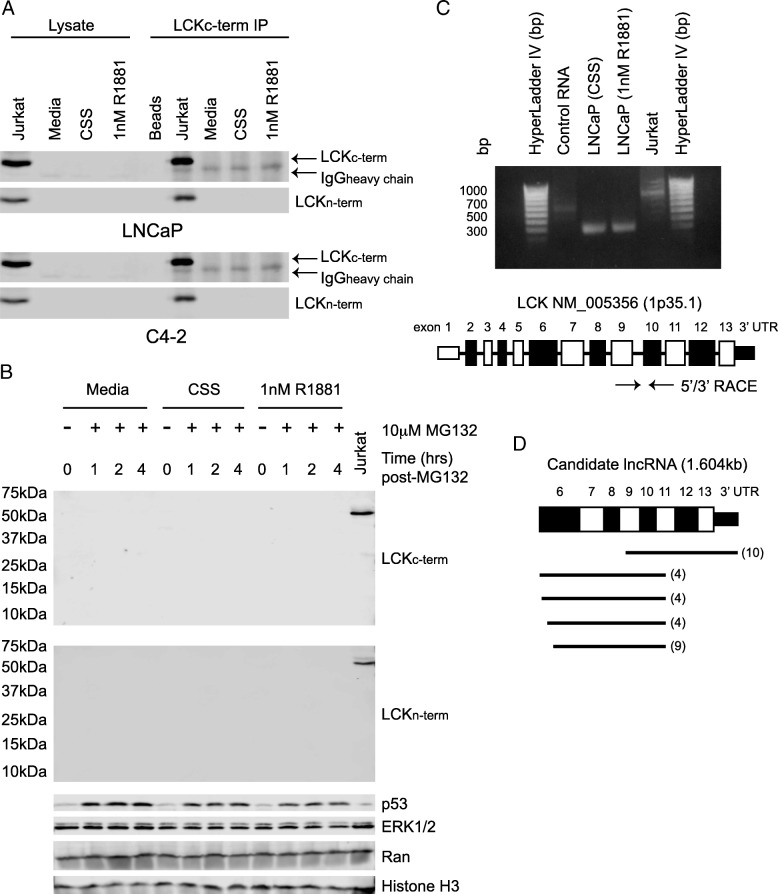
Discovery of a novel lncRNA in prostate cancer cells. **a** LCK protein expression in PCa cells in response to serum starvation or hormone supplementation. LCK protein was immunoprecipitated from 1 mg cell lysate from LNCaP, C4–2, and Jurkat cells cultured in the appropriate growth media for 48 h and treated with 1 nM R1881 or starved of hormone for 24 h, separated by 10% SDS-PAGE, and immunoblotted with LCK antibodies targeting the amino- and carboxy-terminals. Representative blots are shown, *n* = 3. **b** Inhibition of the proteasome does not reveal LCK protein expression. LNCaP cells were seeded and treated with 10 μM MG132 in the presence or absence of R1881 for the indicated timepoints. Cell lysates were blotted for LCK (c-terminal), LCK (n-terminal), p53, ERK1/2, Ran, and Histone H3. Representative blots are shown, *n* = 3. **c** 5′/3′ RACE was performed to determine the sequence of a candidate RNA species. Representative gel is shown, *n* = 3. LNCaP cells were grown in the absence or presence of 1 nM R1881 for 24 h. RNA was collected using the Qiagen RNeasy kit. **d** 5′/3′ RACE identified a candidate lncRNA spanning exon 6 through the 3’UTR of LCK

We previously performed a kinome screen in LNCaP cells grown in the presence or absence of androgen with a panel of shRNAs that targeted the kinome in order to discover potential regulators of growth [18]. The screen identified LCK as a potential positive regulator of growth. Since LCK expression is established in the bone marrow and immune system, but not prostate, we first tested for LCK expression in the hormone-sensitive LNCaP and castration-resistant C4–2 cells by western blotting. We were unable to detect LCK protein expression in LNCaP and C4–2 cells with standard western blots (Fig. 1a). To improve our sensitivity to detect LCK protein, we immunoprecipitated using an antibody that recognized the carboxy-terminal of the protein (LCK^c-term^) (Fig. 1a). As expected, immunoblots showed that two independent LCK antibodies, LCK^c-term^ and an amino-terminal LCK antibody (LCK^n-term^), detected the 56 kDa protein in the Jurkat control cells, which express full-length LCK (FL-LCK). Surprisingly, LCK protein was not detected in LNCaP or C4–2 cells under FBS, CSS, or 1 nM R1881 conditions, suggesting that LCK protein may not be expressed in these cells.

While it has been reported that LCK protein in T-cells has an approximate 20-30 h half-life [21], several Src family members are downregulated following activation as a result of ubiquitination and degradation by the proteasome [22–24]. To determine whether the lack of LCK expression was due to proteasomal degradation, we examined LCK protein levels in the presence of the proteasome inhibitor MG132 (Fig. 1b). The proteasome was successfully blocked since p53, a short-lived protein with a 5-20 min half-life, is elevated in MG132-treated cells [25]. As expected, both LCK^c-term^ and LCK^n-term^ antibodies recognized FL-LCK in Jurkat cells. However, LCK was still not observed at any timepoint examined in LNCaP cells treated with FBS, CSS, or 1 nM R1881. Furthermore, we were unable to detect any truncated forms of LCK when we probed the whole western blot membrane from 75kD to 10kD with either c- or n-terminal LCK antibodies. Thus, these data suggest that LCK protein is not expressed in PCa cells. Therefore, a ncRNA may play a role in the growth effects measured in the kinome screen.

ncRNAs represent over 70% of the human genome and are broadly divided into two main categories: short (< 200 bases) and long (> 200 bases) noncoding transcripts [16]. To determine the full-length sequence of our ncRNA, we carried out 5′ and 3′ RACE (Fig. 1c). Numerous clones (*n* = 31) were selected, purified, and sequenced. The sequence alignment of these clones from the 5′/3′ RACE showed that our ncRNA transcript completely overlapped exon 6 through the 3′ untranslated region (3’UTR) of the *LCK* gene (Fig. 1d). Thus, these data suggest that we have identified a novel lncRNA situated within the *LCK* gene locus that measures 1604 bases in length.

### HULLK is an AR-regulated lncRNA

**Fig. 2:**
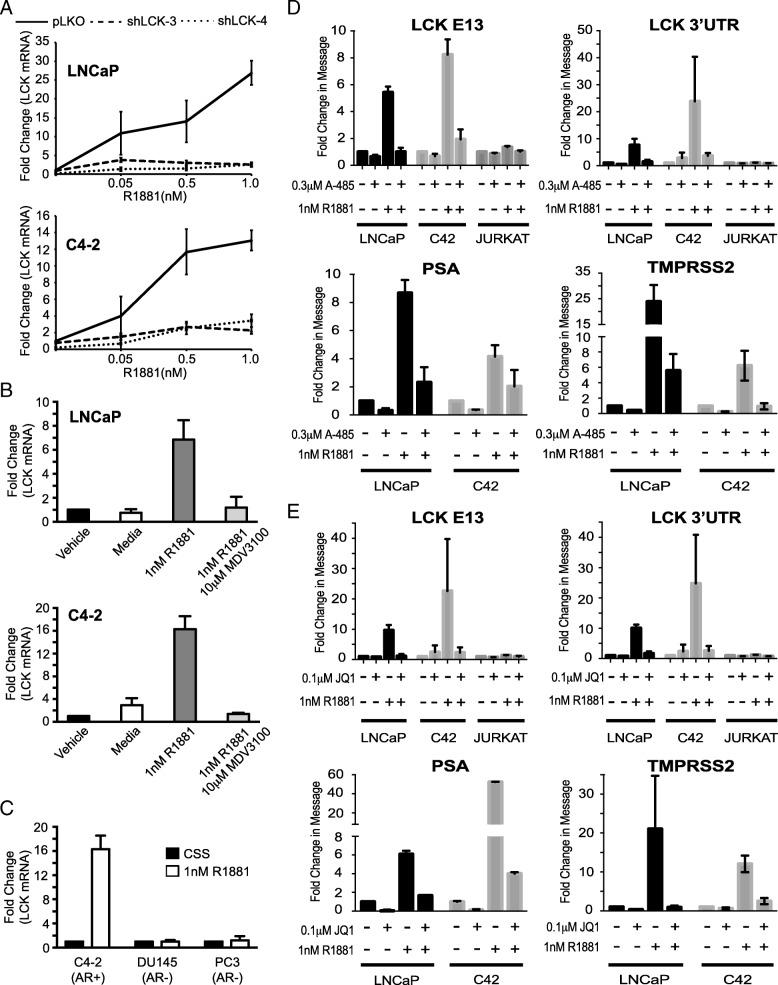
HULLK is an AR-regulated lncRNA. **a** HULLK is positively regulated by androgen, *n* = 3. LNCaP and C42 cells were transduced with two independent shRNAs to LCK and treated with 0-1 nM R1881. RNA was collected on Day 4 and LCK transcript levels were determined using LCK primers targeting the 3’UTR. **b** Enzalutamide blocks the androgen-induced expression of HULLK, *n* = 3. LNCaP and C42 cells were seeded for 48 h, and then, treated with 1 nM R1881 in the presence or absence of 10 μM enzalutamide. RNA was collected 24 h after treatment and LCK transcript levels were determined by qPCR using LCK primers targeting the 3’UTR. **c** HULLK is not upregulated in AR-null PCa cells, *n* = 3. C4–2, DU145, and PC3 cells were seeded in CSS media for 48 h, and then, treated with 1 nM R1881 for 16 h. RNA was collected and LCK transcript levels were determined using LCK primers targeting the 3’UTR. **d** Inhibition of p300 blocks the increase in HULLK expression induced by hormone, *n* = 3. **e** Brd4 inhibition reduces the hormone induction of HULLK, *n* = 3. LNCaP, C42, or Jurkat cells were seeded for 48 h in the appropriate growth media, and then, treated with 1 nM R1881 in the presence or absence of 0.3 μM A-485 or 0.1 μM JQ1. RNA was collected 24 h after treatment and LCK transcript levels were determined by qPCR using LCK primers targeting exon 13 or 3’UTR. PSA and TMPRSS2 transcripts were determined to verify the efficacy of p300 and Brd4 inhibition

To assess whether this lncRNA is modulated by hormone, we quantitated transcript levels of this lncRNA by qPCR using 3’UTR primers in LNCaP and C4–2 cells transduced in the presence of increasing concentrations of R1881 (Fig. 2a). We found that there was a dose-dependent increase in this lncRNA transcripts in response to R1881. This increase was inhibited in the presence of two independent LCK shRNAs in both cell lines. This data suggests that the expression of our lncRNA is regulated by androgen.

We further confirmed this hormone regulation by measuring transcript amounts in LNCaP and C4–2 cells treated with vehicle or R1881 in the presence or absence of the anti-androgen enzalutamide (Fig. 2b). As expected, there was an 8-fold and 12-fold R1881-induced increase in LCK 3’UTR transcript levels in LNCaP and C4–2 cells, respectively. Enzalutamide blocked the hormone-mediated increase in both cell lines. Androgen deprivation influenced message levels negligibly in LNCaP cells, but decreased transcript amounts of this lncRNA by approximately 2.9-fold in C4–2 cells (Fig. 2b*, C4–2 vehicle* vs. *media*). There was no R1881-induced increase in this lncRNA in AR-null DU145 and PC3 cells, validating that the AR regulates the expression of this lncRNA (Fig. 2c). As a result of this regulation, we have chosen to name this lncRNA HULLK for Hormone Upregulated lncRNA within LCK.

AR coregulators have been shown to be influential in the progression of prostate cancer to castration resistance [26]. We noticed that there were binding sites for two AR coactivators (p300 and Brd4) near the HULLK TSS. p300 is known to transactivate the AR in an androgen-dependent [27] and androgen-independent manner [28]. To assess whether p300 is involved in the regulation of HULLK expression by AR, we treated LNCaP, C4–2, and Jurkat cells with the p300 inhibitor A-485 in the presence and absence of 1 nM R1881 and measured HULLK expression by qPCR using two independent LCK primer pairs (Fig. 2d). We found that A-485 alone had negligible effects on HULLK expression. However, the p300 inhibitor significantly opposed the hormone-induced increase in HULLK transcript levels. Analysis of PSA and TMPRSS2 message in the presence of hormone and p300 inhibitor suggests that A-485 was sufficiently blocking its target. We observed similar effects on HULLK expression when we suppressed Brd4 with the BET family small molecule inhibitor JQ1 (Fig. 2e). Brd4 physically associates with the amino-terminal domain of AR, and recruits RNA polymerase II to promote the transcription of target genes [29]. HULLK expression was significantly decreased in the presence of hormone and JQ1, compared to hormone alone. Therefore, these data together indicate that the AR may regulate HULLK expression through the recruitment of p300 and Brd4.

Conversely, we determined whether HULLK modulated AR expression. LNCaP and C4–2 cells were transduced with vector, HULLK, or shRNAs targeting LCK (n-term) (FL-LCK) or LCK (c-term) (HULLK), and AR protein and message levels were examined 48 h after transduction. Knockdown of HULLK did not significantly affect levels of AR protein or mRNA (Additional file 2: Figure S2). Furthermore, AR expression was not notably altered by the overexpression of HULLK (Additional file 2: Figure S2). Therefore, these data suggest that HULLK is an AR-regulated lncRNA that does not reciprocally influence expression of AR itself.

### Characterization of HULLK

**Fig. 3:**
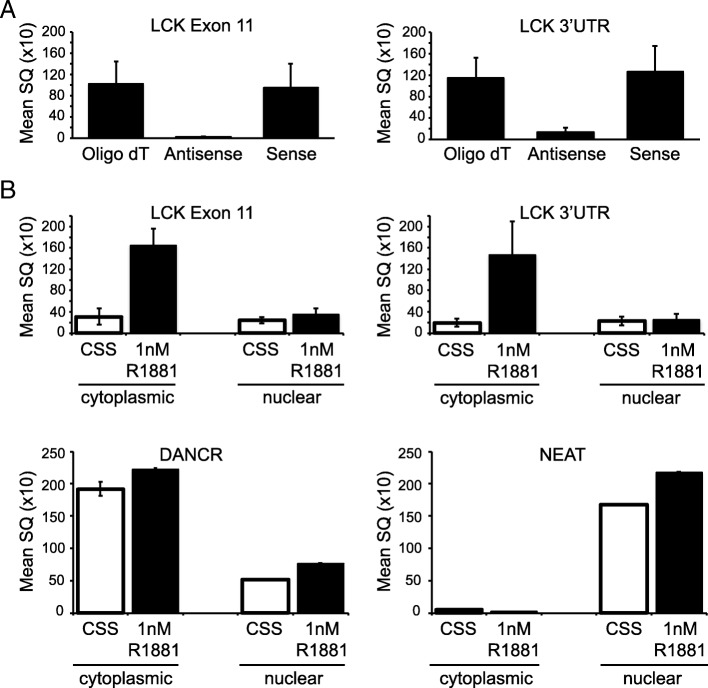
Characterization of HULLK. **a** HULLK is transcribed from the sense strand of DNA. LNCaP cells were seeded for 48 h and treated with 1 nM R1881 for 24 h. RNA was collected and cDNA was synthesized using strand-specific primers. qPCR was performed with two independent LCK primer sets that amplify HULLK, *n* = 3. **b** HULLK is localized to the cytoplasm. LNCaP cells were seeded for 48 h and treated with 1 nM R1881 for 24 h. Whole cell lysates were separated into cytoplasmic and nuclear fractions by centrifugation. RNA was collected from each fraction and cDNA was synthesized using the iScript cDNA synthesis kit. qPCR was performed with two independent LCK primer sets that amplify HULLK, and cytoplasmic and nuclear control genes, *n* = 3

Since lncRNAs can be derived from many different genomic locations and transcribed from either DNA strand, we utilized strand-specific qPCR to determine which DNA strand HULLK is transcribed from. Total RNA was collected from LNCaP cells grown in complete media, and cDNA template was synthesized using Oligo (dT)16, antisense strand-specific, or sense strand-specific primers (Additional file 1: Figure S1). qPCR was performed with LCK Exon 11 and LCK 3’UTR primer pairs (Additional file 1: Figure S1). Amplification of PCR products from both LCK primer pairs was only observed with the Oligo (dT)16 and sense strand-specific cDNA templates (Fig. 3a). However, there was no significant PCR amplification from either LCK primer pair when antisense strand-specific cDNA was used as the template. These data indicate that HULLK is transcribed from the sense strand of DNA.

Some lncRNAs show preferential accumulation in the cytoplasm or nucleus, while other lncRNAs are equally expressed in both compartments. To determine the intracellular localization of HULLK, we collected total RNA to synthesize cDNA from cytoplasmic and nuclear fractions of LNCaP cells starved of hormone or exposed to 1 nM R1881 for 24 h and carried out qPCR with LCK E11 and LCK 3’UTR primers to amplify HULLK. Amplification of a predominantly cytoplasmic lncRNA DANCR [30] and nuclear lncRNA NEAT1 [31] showed that transcript levels under both conditions were substantially higher in the cytoplasm for DANCR and nucleus for NEAT1, indicating efficient cellular fractionation (Fig. 3b). We observed that hormone dramatically increased LCK E11 and LCK 3’UTR transcript levels approximately 5.3-fold and 7.6-fold, respectively, in the cytoplasmic fraction, whereas the nuclear transcript amounts were approximately equivalent to the cytoplasmic CSS condition and not influenced by androgens. These results indicate that HULLK is localized to the cytoplasm.

### HULLK expression in PCa

**Fig. 4:**
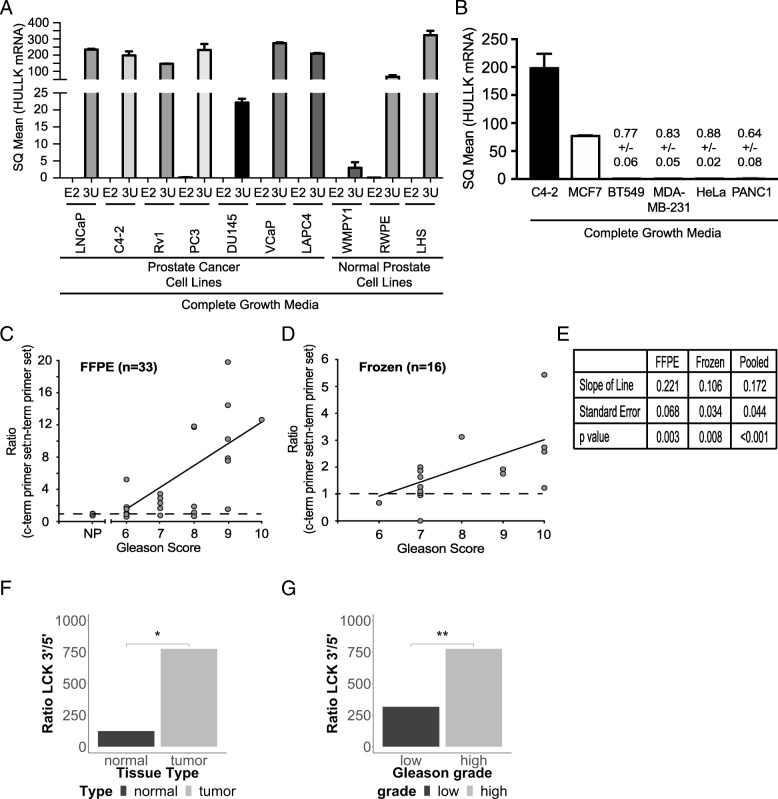
HULLK expression in prostate cancer. **a** Expression of HULLK in a panel of PCa and normal prostate epithelial cell lines cultured in complete growth media. qPCR was performed with two independent LCK primer sets targeting exon 2 (E2) and the 3’UTR (3 U), *n* = 3. **b** HULLK expression in C4–2, MCF7, BT549, MDA-MB-231, HeLa, and PANC1 cell lines grown in complete growth media. qPCR was performed with LCK primers targeting the 3’UTR, *n* = 3. **c** A significant positive correlation between HULLK expression and PCa grade. 33 FFPE tissue samples were obtained from the Biorepository and Tissue Research Facility at the University of Virginia. These samples represent biopsies and surgical resections collected in 2016 from PCa patients presenting with Gleason Score 6–10. Normal and cancerous regions were demarcated and 1.5 mm punches were collected. ddPCR was performed on the QX200 droplet digital PCR system (Bio-Rad). **d** 16 fresh-frozen tissue samples from Gleason Score 6–10 PCa patients were acquired from Dr. Ganesh Raj from the University of Texas Southwestern Medical Center. These samples were surgically resected from May to December in 2017, placed in RNALater (ThermoFisher), and frozen immediately. **e** Statistical analysis of the association between the LCK E13 to LCK E4 ratio and grade was performed using linear regression models, following transformation to the log scale. The analyses were conducted separately for frozen and FFPE samples, with a test for interaction used to test whether the association between the E13 to E4 ratio and grade was the same in frozen samples as in FFPE samples. **f** HULLK expression in normal and tumor and **g** low Gleason [scores 6 and 7(3 + 4)] and high Gleason score [scores 7(4 + 3), and 8–10] in the PRAD TCGA cohort

Studies have shown that lncRNAs are abnormally expressed in many cancers, including prostate [32]. Since HULLK was previously unannotated, little is known about its expression pattern. We first discovered HULLK in PCa cells, and thus, we surveyed a panel of PCa and normal prostate epithelial cell lines cultured in their corresponding growth media for HULLK expression by qPCR (Fig. 4a). Since the sequence of HULLK overlapped LCK exon 6 through the 3’UTR, we exploited the fact that HULLK lacked exons 1–5 to distinguish HULLK expression from FL-LCK. Therefore, we utilized two LCK primer pairs – LCK 3’UTR and LCK exon 2 (LCK E2). LCK 3’UTR primers should amplify HULLK as well as FL-LCK; however, LCK E2 primers should only detect FL-LCK. Analysis of seven PCa cell lines (LNCaP, C4–2, CWR22Rv1, PC3, DU145, VCaP, LAPC4) and three normal prostate cell lines (WMPY1, RWPE, LHS) revealed that HULLK is expressed to varying degrees in all cell lines examined, as PCR amplification was only detected with the LCK 3’UTR primers and not the LCK E2. As a control for primer efficiency, we detected FL-LCK in Jurkat cells with both primer pairs (*data not shown*). These data show that we can successfully detect HULLK employing this qPCR method of two primer pairs targeting two specific regions in LCK – exons 1–5 and exon 6–3’UTR.

We also examined HULLK expression in other cancer tissue types and did not observe any transcripts in cervical (HeLa) or pancreatic (PANC1) cancer cell lines grown under normal serum conditions (Fig. 4b). We did detect HULLK in the ER + AR+ luminal A subtype MCF7 breast cancer (BCa) cell line but not in the triple negative Claudin-low BT549 or MDA-MB-231 BCa cell lines [33].

Applying the two LCK primer pair method, HULLK levels were determined in twenty-six FFPE PCa tissue samples from patients presenting with Gleason score 6–10 disease and seven normal prostate tissue obtained from the University of Virginia Biorepository and Tissue Research Facility (Fig. 4c). We displayed the results as the ratio of SQ mean of the carboxy-terminal primer pairs to the amino-terminal primer pair, where < 1 indicates FL-LCK and > 1 indicates more HULLK. We did not detect HULLK expression in the normal prostate tissue that were examined. However, we found that there was a significant positive correlation between HULLK expression and higher Gleason score. We confirmed this correlation using a second cohort of 16 fresh-frozen PCa tissue from the University of Texas Southwestern (Fig. 4d). In this cohort, we discovered similar results as the first cohort; there was a significant increase in HULLK expression with increased Gleason score. Statistical analyses revealed that pooling the data from the two cohorts does not diminish the significance of this correlation between HULLK levels and Gleason score (Fig. 4e). Finally, we interrogated the PRAD TCGA cohort [19] for HULLK expression and association with clinical correlate. The LCK exon-specific RSEM data for all samples in this cohort was obtained from TSVdb [20], to calculate the 3′/5′ ratio and define HULLK expression as an increase in the 3′/5′ ratio. We found that there was an increase in HULLK expression when comparing normal to tumor and, in tumors only, low Gleason [scores 6 and 7(3 + 4)] to high Gleason score [scores 7(4 + 3), and 8–10]. Interestingly, we found very high 3′/5′ ratios in the PRAD TCGA dataset. However, applying a multivariate Cox Proportional Hazards model, controlling for age and Gleason grade, we were unable to determine if HULLK expression alone was significantly associated with shorter time to biochemical recurrence (data not shown). These data strongly indicate that HULLK is expressed in PCa patients and upregulated with advanced disease.

### HULLK positively regulates PCa cell growth

**Fig. 5:**
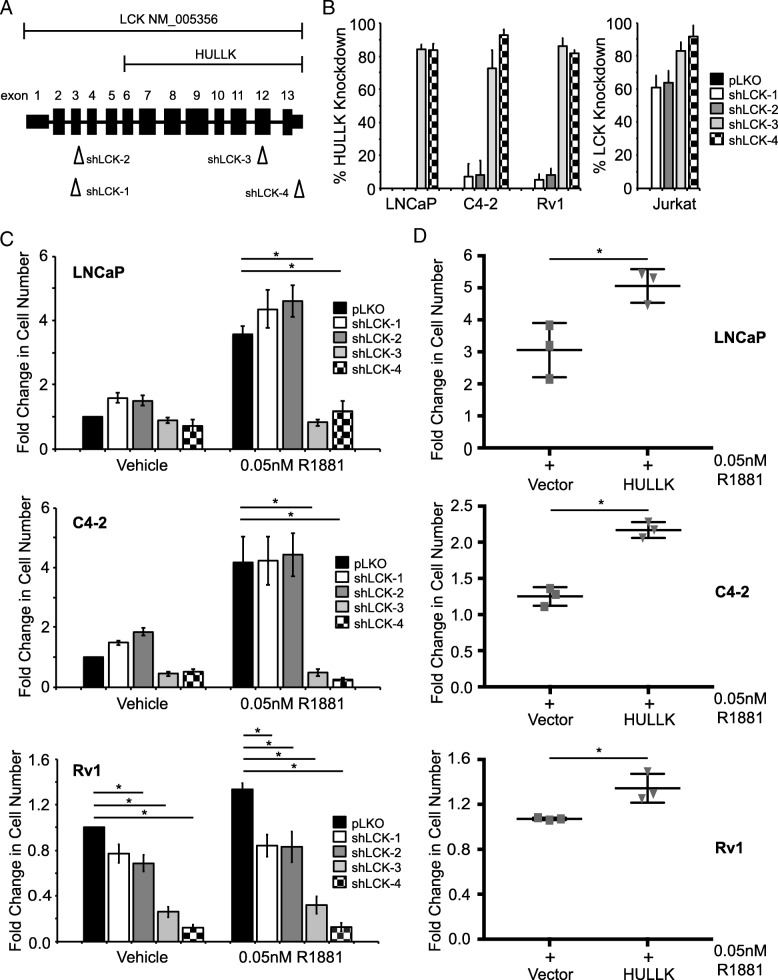
HULLK positively regulates prostate cancer cell growth. **a** Gene structure of LCK and location of each shRNA targeting LCK. **b** Percent knockdown of HULLK or LCK message, *n* = 3. MISSION shRNAs targeting human LCK (NM_005356) and the pLKO vector control were purchased from ThermoFisher. Each shRNA was validated for efficiency of LCK or HULLK knockdown in Jurkat and PCa cells by qPCR. **c** CyQuant Assay measured DNA content as a surrogate for cell number 7 days after shRNA transduction. Growth was compared to untreated empty vector control and the values were averaged across biological replicates. Error bars represent standard error of the mean. The relative effect of four independent shRNAs on cell growth in LNCaP, C42, and Rv1 cells in the absence or presence of 0.05 nM R1881, *n* = 3. **d** The relative effect of HULLK overexpression on cell growth in LNCaP, C42, and Rv1 cells in the absence or presence of 0.05 nM R1881, *n* = 3. Statistical analysis was performed using ANOVA and Tukey test

To explore the functional role of HULLK in PCa, we examined the effects of HULLK knockdown on PCa cell growth. LNCaP, C4–2, and Rv1 cells were transduced with lentiviral particles expressing four independent shRNAs specific for LCK or pLKO empty vector control in the presence or absence of 0.05 nM R1881. Two shRNAs were targeting the carboxy-terminal of LCK (shLCK-3 and shLCK-4) and should decrease HULLK levels; and two shRNAs were directed toward the amino-terminal (shLCK-1 and shLCK-2) and should not affect HULLK expression (Fig. 5a). We confirmed by qPCR that shLCK-3 and shLCK-4 decreased HULLK expression by 70–90% in LNCaP, C4–2, and Rv1 cells (Fig. 5b). shLCK-1 and shLCK-2 had little to no effect on HULLK expression in these three cell lines. As a control, all four shRNAs efficiently knocked LCK down 60–90% in Jurkat cells. Seven days following viral transduction, we measured cellular growth using CyQuant, which uses DNA content as a surrogate for cell number. In the absence of hormone, the four shRNAs had no dramatic effects on growth in LNCaP or C4–2 cells (Fig. 5c). However, there was a statistically significant decrease in cell growth in Rv1 cells with shLCK-2, shLCK-3, and shLCK-4. In the presence of 0.05 nM R1881, the carboxy-terminal shRNAs significantly inhibited growth of LNCaP, C4–2, and Rv1 cells compared to the pLKO control cells. While the amino-terminal shRNAs did not have any significant effects on growth in LNCaP or C4–2 cells, there was a notable proliferative decrease in Rv1 cells. These data suggest that HULLK may drive PCa cell growth.

We performed the complement experiment of evaluating the effects of overexpressing HULLK on cell growth. LNCaP, C4–2, and Rv1 cells were transduced with lentivirus expressing either vector or HULLK constructs in the presence or absence of 0.05 nM R1881, and proliferation was calculated 7 days following viral transduction. Overexpression of HULLK did not provide a growth advantage over vector-expressing cells in androgen-deprived media (data not shown). We did observe the expected increase in cell growth in all vector-expressing control cells exposed to R1881 (Fig. 5d). However, hormone significantly increased proliferation in HULLK-overexpressing cells compared to vector control cells, suggesting that there may be a greater sensitivity to hormone when HULLK is overexpressed in PCa cells. Together, these data indicate that HULLK is a positive regulator of PCa cell growth and may help drive CRPC by increasing sensitivity to hormone.

## Discussion

One in 41 men diagnosed with PCa will die from the disease (cancer.org). While ADT is the current initial treatment for advanced PCa, eventually all men diagnosed with PCa will develop incurable CRPC. Therefore, there exists a serious need for more effective therapies for the treatment of advanced PCa, and that requires a more complete understanding of PCa development and progression. Herein we describe a previously unannotated lncRNA we have named HULLK, for Hormone Upregulated lncRNA within LCK, that functions as a positive regulator of PCa cells and the expression of which correlates with high grade PCa.

Genome sequencing has led to the surprising discovery that protein-coding RNAs only make up approximately 2% of the human genome, while ncRNAs represents 70–90% [34]. Once believed to be transcriptional noise, ncRNAs are now associated with many normal biological processes, including transcriptional and translational regulation, chromatin modification, and cell cycle regulation. Furthermore, accumulating evidence supports vital roles in cancer initiation, development, and progression for ncRNAs [35, 36], which are generically divided into two groups: small and long noncoding transcripts. While small ncRNAs, especially microRNAs, have been well-documented to play important roles in human disease by regulating the expression of target mRNA, lncRNAs are only beginning to be investigated and scrutinized. Chinnaiyan and colleagues sequenced the transcriptomes of PCa tissue samples and cell lines to find that roughly 25% corresponded to lncRNAs, suggesting that they may be important in cellular biology and carcinogenesis [1]. Even though thousands of lncRNAs have been annotated in the human genome, only several functional lncRNAs in PCa have been fully characterized and experimentally validated [1, 15, 37, 38].

Here, we identified a novel lncRNA in PCa from a high-throughput RNAi screen to uncover potential regulators of PCa cell growth [18]. LCK was identified from the shRNA screen as a positive regulator of PCa cellular proliferation. However, LCK protein expression was never detected. Immunoprecipitation and Western analyses revealed that LCK could only be observed in control Jurkat cells and not LNCaP or C4–2 PCa cell lines. The inability to recognize LCK in PCa cells was not due to the short LCK half-life, as the proteasome inhibitor MG132 did not facilitate the detection of LCK protein. The open reading frame (ORF) finder from the National Center for Biotechnology Information predicted that there were one long and two short ORFs in the HULLK sequence. According to the GenScript Codon Bias tool, the two short ORFs have low codon adaptation indices (CAI) (< 0.8), suggesting poor expression. The long ORF, which is in frame with LCK and corresponds to the kinase domain, has a CAI (0.83) similar to LCK exons 1–5, suggesting that this ORF calculated to produce a 28.59 kDa protein has an equal chance of being expressed as exons 1–5 of LCK. Furthermore, data from the FANTOM Project suggested that there was a TSS near LCK exon 12, resulting in an estimated 13 kDa truncated LCK protein [39]. Using an antibody raised against tyrosine 505 in exon 13 (kinase domain) of human LCK, we were unable to detect the predicted 28.59 kDa or 13 kDa LCK protein or any other shorter LCK isoforms. This antibody did recognize LCK in Jurkat cells. These data suggested that LCK protein was not expressed in PCa cells. Our data are consistent with the ChIP-seq dataset from the ENCODE Project, where the H3K4me3 peaks were highly enriched at active promoters near TSSs in Jurkat cells [40]. Therefore, the shRNAs utilized in the kinome screen may have targeted a noncoding RNA within the *LCK* gene locus.

We utilized 5′/3′ RACE to uncover a novel noncoding transcript that aligned with the *LCK* gene and completely overlapped exons 6–13 and 3’UTR on the sense strand of DNA. All of the clones examined from the 3′ RACE showed the same end, but several clones from the 5′ RACE varied in the ends by roughly 5–20 nucleotides. The presence of a 5′-3′ exoribonuclease in the PCR reaction likely account for these differences in 5′ ends.

Ning and colleagues reanalyzed the dataset from the Necsulea et al study [41] and reported that 29.3% of 24,793 annotated lncRNAs overlapped protein-coding genes [42]. The overlapping lncRNAs were categorized into five main groups based on DNA strand: overlapping on opposite strands (5′-regions overlap, 3′-regions overlap, embedded pairs) and overlapping on same strand (5′-regions overlap with 3′-regions, embedded pairs). The embedded pairs on either strand represented 76.4% of all annotated overlapping lncRNA-protein-coding pairs. The majority of the overlaps (~ 93%) occurred on the opposite DNA strand. However, lncRNAs embedded in a protein-coding gene on the same DNA strand only amounted to 5.6%. Similar to the fact that overlapping genes tend to be coexpressed [43], lncRNA-protein-coding pairs exhibited an overall positive expression correlation, with the Spearman coefficient for same strand overlaps being greater than opposite strand overlaps [42]. These observations suggested that the HULLK-LCK gene pair may be rare, since they overlapped on the same strand and the protein-coding partner is not expressed in PCa cells.

The cellular compartment in which a particular lncRNA is localized may be used as a cue to its functions. Nuclear lncRNAs can facilitate epigenetic regulation through the binding of chromatin and chromatin modifying proteins [44], whereas cytoplasmic lncRNAs can influence several cellular processes, including protein degradation and transcription [45]. Much less is known about cytoplasmic lncRNAs compared to their nuclear counterparts. Our work on HULLK augments the growing list of cytoplasmic lncRNA functions. We explored the functional role of HULLK in PCa by knocking down HULLK with two independent shRNAs in LNCaP, C4–2, and Rv1 cells. There was a significant decrease in cell growth in the absence and presence of androgens when HULLK was depleted from the cell. HULLK overexpression resulted in a modest but consistent increase in cellular proliferation in response to hormone. These results suggested that HULLK is oncogenic in nature, similar to other lncRNAs in PCa, including PCAT-1 [45], PCA3 [3], and SChLAP1 [46].

The expression of lncRNAs typically has been cell-type and tissue specific [1]. Furthermore, lncRNAs that overlap protein-coding genes showed higher tissue specificity than non-overlapping lncRNA-protein-coding gene pairs [42]. Our data were mainly consistent with the tissue specificity of lncRNAs, as we observed HULLK expression in a panel of PCa cell lines and tissue. We did not quantitate any HULLK transcripts above transcriptional noise in cervical or pancreatic cancer cell lines. However, we did discover HULLK transcripts in MCF7 cells and not BT549 or MDA-MB-231 cells. While all three of the BCa lines are reported to express AR [47, 48], in our laboratory we only detect AR protein levels in MCF7 and not BT549 or MDB-MB-231 cells (Additional file 3: Figure S3). Furthermore, HULLK expression increases in response to 1 nM R1881 in MCF7 cells, but not BT549 or MDA-MB-231 cells (Additional file 3: Figure S3). In addition to the prostate specificity of HULLK, these data also suggest that HULLK may be expressed in other tissues where AR is active. Akin to PCGEM1 [49], CTBP1-AS [11], and PCA3 [3] lncRNAs, HULLK was upregulated in response to androgen. More importantly, expression of HULLK was significantly upregulated in high-grade PCa specimens in three cohorts, including the PCa TCGA.

Since LCK-expressing lymphocytes infiltrate prostate tumors [50], we exploited the fact that HULLK lacked *LCK* exons 1–5, and used the ratio of c-terminal LCK primers:n-terminal primers (HULLK:FL-LCK) to distinguish HULLK expression from *LCK*. We noticed that the ratios for the FFPE samples were higher than the fresh frozen tissue. These ratios could be influenced by differences in tissue collection and composition. For the FFPEs, the extent of infiltrating lymphocyte contamination was reduced by demarcating portions of tumor from surrounding lymphocyte-containing stroma. Decreasing the stromal contribution would diminish the amount of LCK, and thus, increase the ratios of HULLK:LCK. Even with the compounded presence of lymphocytes in the fresh frozen cohort, HULLK expression still displayed a significant upregulation with increasing Gleason score. Examining the TCGA data for the 3′/5′ ratio of LCK validated this observation, which showed increasing HULLK expression in tumor compared to normal, and within tumors, with higher Gleason score compared to low Gleason score. The same finding of HULLK expression correlating with Gleason score in three independent cohorts, when considered with the experimental data that HULLK over expression increases growth and HULLK knockdown decreases PCa growth in androgen-dependent and castration-resistant PCa lines, strongly suggests that HULLK expression is associated with aggressive disease and may be a driver of PCa.

We explored a potential mechanisms for growth stimulation by HULLK. Since HULLK is embedded in the *LCK* gene locus, we first hypothesized that HULLK may be regulating cell growth through the modulation of the expression of Src family kinases. Knockdown of HULLK with shRNAs revealed that the levels of protein and message of each Src family member examined (Blk, Fgr, Frk, Fyn, Hck, Lyn, Src, and Yes) were not significantly altered, suggesting that HULLK may not affect cell proliferation through the regulation of Src family kinase expression (data not shown).

lncRNAs can exert their effects locally by regulating the expression of neighboring genes in *cis* or distantly in *trans* [51]. There are two genes immediately 5′ (Eukaryotic translation initiation factor 3 subunit I, EIF3I) and 3′ (histone deacetylase 1, HDAC1) of HULLK with reported biological functions that may influence cell growth. EIF3I plays a crucial role in the initiation of protein synthesis, whereas HDAC1 interacts with the retinoblastoma tumor-suppressor protein to control cell proliferation and differentiation [52]. To determine whether the effects on cell growth mediated by HULLK were a result of the regulation of gene expression in *cis*, we measured the transcript levels of EIF3I and HDAC1 in HULLK-depleted PCa cells. We found that HULLK knockdown did not dramatically change the amounts of message of either gene, suggesting that the regulation of gene expression in *cis*, at least for these two genes, may not account for the HULLK-mediated effects on cell growth (data not shown). While we did not see any significant alterations in these genes at the RNA level, we cannot rule out the possibility that HULLK may be affecting these genes at the protein level. lncRNAs can function as scaffolds or sponges, thereby influencing translation efficiency, cellular localization, or protein stability [51]. More experiments will need to be performed to determine whether HULLK is regulating cell proliferation by functioning as a molecular scaffold or sponge. Specifically, defining and comparing the proteins and/or nucleic acids complexed with HULLK under hormone-sensitive and CRPC should aid in the elucidation of the mechanism of growth stimulation by HULLK, thereby clarifying HULLK’s oncogenic characteristics.

## Conclusions

In this study, we show that there is a novel lncRNA completely overlapping exons 6–13 and 3’UTR of the *LCK* gene on 1p35.1. We establish that this lncRNA is regulated by androgens and the AR, and as such, we have named this lncRNA “HULLK” for Hormone Upregulated lncRNA within *LCK*. We reveal that HULLK is localized to the cytoplasm and found on the sense strand within the LCK gene. HULLK is expressed in PCa cell lines and upregulated as PCa progresses to metastatic disease. While the full repertoire of HULLK functions remains to be elucidated, we demonstrate that HULLK functions as an oncogene to positively regulate PCa cell proliferation. Thus, our data enhances our understanding of lncRNA biology and may assist in the development of additional biomarkers or more effective therapeutic targets for advanced PCa.

## Additional files

Additional file 1: **Figure S1.** PCR Primer Sequences. (A) HULLK cloning primers. (B) 5′/3′ RACE primers. (C) Strand-specific PCR primers. (D) lncRNA localization primers (PDF 589 kb)

Additional file 2: **Figure S2.** HULLK expression does not affect AR expression. (A) Expression of AR following HULLK knockdown. LNCaP and C4–2 cells were transduced with vector, shLCK (n-term), or shLCK (c-term) in the appropriate growth media, and whole cell lysates were collected 48 h later and blotted for AR21 and ERK1/2, *n* = 3. (B) Expression of AR following HULLK overexpression. LNCaP and C4–2 cells were transduced with vector or HULLK in the appropriate growth media, and whole cell lysates were collected 48 h later and blotted for AR21 and α-tubulin, *n* = 3. RNA was collected and AR transcript levels were determined using AR primers targeting the DNA binding domain, *n* = 3. (PDF 26336 kb)

Additional file 3: **Figure S3.** Regulation of HULLK by the AR in breast cancer cells. (A) Expression of AR in breast cancer cell lines. LNCaP, MCF7, BT549, and MDA-MD-231 cells were seeded in the appropriate growth media, and whole cell lysates were collected 48 h later and blotted for AR21 and ERK1/2. (B) HULLK expression increases in response to hormone. MCF7, BT549, and MDA-MB-231 cells were seeded in CSS media for 48 h, and then, treated with 1 nM R1881 for 16 h. RNA was collected and LCK transcript levels were determined using LCK primers targeting the 3’UTR, *n* = 3. (PDF 1434 kb)

## Data Availability

All data generated or analyzed during this study are included in this published article and its additional files.

## Abbreviations

ADT: Androgen deprivation therapy
BCa: Breast cancer
BET: Bromodomain and extra-terminal
CRPC: Castration-resistant prostate cancer
CTBP1-AS: C-terminal binding protein 1-antisense
DANCR: Differentiation antagonizing non-protein coding RNA
ddPCR: Droplet digital PCR
EIF3I: Eukaryotic translation initiation factor 3 subunit I
EMT: Epithelial-to-mesenchymal transition
ENCODE: Encyclopedia of DNA elements
FANTOM: Functional annotation of the mammalian genome
FFPE: Formalin-fixed paraffin-embedded
GAS5: Growth arrest-specific 5
HDAC1: Histone deacetylase 1
HOTAIR: HOX transcript antisense RNA
HULLK: Hormone-upregulated lncRNA within *LCK*
LCK: Lymphocyte-specific protein tyrosine kinase
lncRNA: Long noncoding RNA
lncRNA-ATB: long noncoding RNA Activated by Transforming Growth Factor-Beta
MDM2: Mouse double minute 2 homolog
ncRNA: noncoding RNA
NEAT1: Nuclear-enriched abundant transcript 1
NP: Normal prostate
PCa: Prostate cancer
PCA3: Prostate Cancer Antigen 3
PCAT: Prostate cancer-associated transcript
PRUNE2: Prune homolog 2
qPCR: Quantitative PCR
RACE: Rapid amplification of cDNA ends
SChLAP1: Second chromosome locus associated with prostate-1
shRNA: short-hairpin RNA
TMPRSS2: Transmembrane protease, serine 2
TSS: Transcriptional start site
