# Supplementary material for: Discovery of a novel long noncoding RNA overlapping the LCK gene that regulates prostate cancer cell growth
Source: Mol Cancer. 2019 Jun 28;18:113. doi: 10.1186/s12943-019-1039-6 (PMC6598369; doi:10.1186/s12943-019-1039-6)
Supplement: Supplementary file 1 — Figure S1. PCR Primer Sequences. (A) HULLK cloning primers. (B) 5′/3′ RACE primers. (C) Strand-specific PCR primers. (D) lncRNA localization primers (PDF 589 kb) [file 12943_2019_1039_MOESM1_ESM.pdf]

A.

**HULLK Cloning Primers**	
HULLK-attB1-Forward	5’-GGGGACAAGTTTGTACAAAAAAGCAGGCTTCC TGGTTCTTCAAGAACCTGAG-3’
HULLK-attB1-Start-Forward	5‘-GGGGACAAGTTTGTACAAAAAAGCAGGCTTCG AAGGAGATAGAACCATGGCTGGTTCTTCAAGAAC CTGAG-3’
HULLK-attB2-Reverse	5’-GGGGACCACTTTGTACAAGAAAGCTGGGTCTC ATCAACAGACATTTATTGAACTC-3’
HULLK-attB2-Stop-Reverse	5’-GGGGACCACTTTGTACAAGAAAGCTGGGTCCT ATCATCAACAGACATTTATTGAACTC-3’

B.

**5’ RACE Primers**	
LCK-GSP1-Reverse	5’-CTTCGTGTGCCCGTTGTAGTA-3’
LCK-GSP2-Reverse	5’-CCCGAAGGTCACGATGAATAT-3’
**3’ RACE Primers**	
LCK-GSP1-Forward	5’-TACCAACTCATGAGGCTGTGC-3’

C.

LCK Sense-Strand Primer	5’-CAGACATTTATTGAACTCCTGA-3’
LCK Anti-Sense-Strand Primer	5’-ATCGTTTTCACTGTCGGT-3’

D.

**LCK Primers**:	
**LCK Exon 2**	(F) 5’-GTGTGAGAACTGCCATTATC-3’_(R) 5’-AGAGCCATTTCGGATGAG-3’
**LCK Exon 4**	(F) 5’-CAACCTGGTTATCGCTCT-3’_(R) 5’-CCTTCTCAAAGCCCAGAT-3’
**LCK Exon 11**	(F) 5’-ATGGCATTCATTGAAGAGC-3’_(R) 5’-GTCAGACACCAGAATGTTG-3’
**LCK Exon 13**	(F) 5’-GGAGCTGTACCAACTCAT-3’_(R) 5’-CAGGTAGTCAAAGGTGGG-3’
**LCK 3’UTR**	(F) 5’-ATCCAGAAGTTCCTCAAG-3’_(R) 5’-TTACAACAGTCATCAACAG-3’
**lncRNA Primers**:	
**DANCR**	(F) 5’-CGGAGGTGGATTCTGTTA-3’_(R) 5’-GTGTAGCAAGTCTGGTGA-3’
**NEAT**	(F) 5’-GGTCTGAGGAGTGATGTG-3’_(R) 5’-AAGCGTTGGTCAATGTTG-3’

Figure S1
