# Supplementary figures and images for: Discovery of a novel long noncoding RNA overlapping the LCK gene that regulates prostate cancer cell growth
Source: Mol Cancer. 2019 Jun 28;18:113. doi: 10.1186/s12943-019-1039-6 (PMC6598369; doi:10.1186/s12943-019-1039-6)

A.

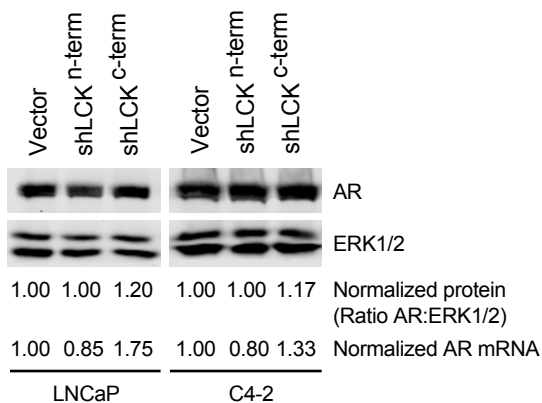

B.

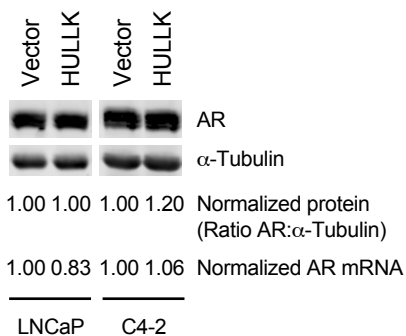

Figure S2

Supplement: Supplementary file 2 — Figure S2. HULLK expression does not affect AR expression. (A) Expression of AR following HULLK knockdown. LNCaP and C4–2 cells were transduced with vector, shLCK (n-term), or shLCK (c-term) in the appropriate growth media, and whole cell lysates were collected 48 h later and blotted for AR21 and ERK1/2, n = 3. (B) Expression of AR following HULLK overexpression. LNCaP and C4–2 cells were transduced with vector or HULLK in the appropriate growth media, and whole cell lysates were collected 48 h later and blotted for AR21 and α-tubulin, n = 3. RNA was collected and AR transcript levels were determined using AR primers targeting the DNA binding domain, n = 3. (PDF 26336 kb) [file 12943_2019_1039_MOESM2_ESM.pdf]

A.

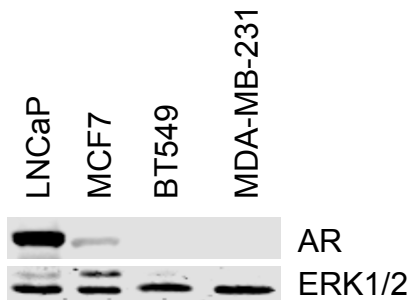

B.

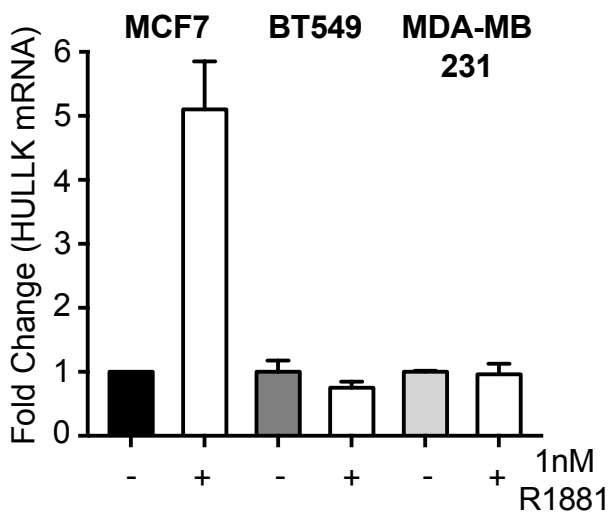

Figure S3

Supplement: Supplementary file 3 — Figure S3. Regulation of HULLK by the AR in breast cancer cells. (A) Expression of AR in breast cancer cell lines. LNCaP, MCF7, BT549, and MDA-MD-231 cells were seeded in the appropriate growth media, and whole cell lysates were collected 48 h later and blotted for AR21 and ERK1/2. (B) HULLK expression increases in response to hormone. MCF7, BT549, and MDA-MB-231 cells were seeded in CSS media for 48 h, and then, treated with 1 nM R1881 for 16 h. RNA was collected and LCK transcript levels were determined using LCK primers targeting the 3’UTR, n = 3. (PDF 1434 kb) [file 12943_2019_1039_MOESM3_ESM.pdf]
